# *Drosophila* Bitter Taste(s)

**DOI:** 10.3389/fnint.2015.00058

**Published:** 2015-11-25

**Authors:** Alice French, Moutaz Ali Agha, Aniruddha Mitra, Aya Yanagawa, Marie-Jeanne Sellier, Frédéric Marion-Poll

**Affiliations:** ^1^Evolution, Génomes, Comportement & Ecologie, CNRS, IRD, Université Paris-Sud, Université Paris-SaclayGif-sur-Yvette, France; ^2^Research Institute for Sustainable Humanosphere, Kyoto UniversityUji City, Japan; ^3^AgroParisTechParis, France

**Keywords:** taste, insects, aversive, pheromones, electrophysiology, behavior

## Abstract

Most animals possess taste receptors neurons detecting potentially noxious compounds. In humans, the ligands which activate these neurons define a sensory space called “bitter”. By extension, this term has been used in animals and insects to define molecules which induce aversive responses. In this review, based on our observations carried out in *Drosophila*, we examine how bitter compounds are detected and if bitter-sensitive neurons respond only to molecules bitter to humans. Like most animals, flies detect bitter chemicals through a specific population of taste neurons, distinct from those responding to sugars or to other modalities. Activating bitter-sensitive taste neurons induces aversive reactions and inhibits feeding. Bitter molecules also contribute to the suppression of sugar-neuron responses and can lead to a complete inhibition of the responses to sugar at the periphery. Since some bitter molecules activate bitter-sensitive neurons and some inhibit sugar detection, bitter molecules are represented by two sensory spaces which are only partially congruent. In addition to molecules which impact feeding, we recently discovered that the activation of bitter-sensitive neurons also induces grooming. Bitter-sensitive neurons of the wings and of the legs can sense chemicals from the gram negative bacteria, *Escherichia coli*, thus adding another biological function to these receptors. Bitter-sensitive neurons of the proboscis also respond to the inhibitory pheromone, 7-tricosene. Activating these neurons by bitter molecules in the context of sexual encounter inhibits courting and sexual reproduction, while activating these neurons with 7-tricosene in a feeding context will inhibit feeding. The picture that emerges from these observations is that the taste system is composed of detectors which monitor different “categories” of ligands, which facilitate or inhibit behaviors depending on the context (feeding, sexual reproduction, hygienic behavior), thus considerably extending the initial definition of “bitter” tasting.

## Introduction

In humans, bitter taste is defined as a sensation associated with the perception of potentially toxic molecules such as alkaloids, which induce innate aversive reactions (Ventura and Worobey, 2013). Innate aversions can be subsequently reversed, and bitter tasting foods can even become appealing for example when post-ingestive effects are positive either physiologically or socially (Calabrese, 2008). Molecular studies support the view that bitter taste is mediated in vertebrates by specific receptor proteins Tas2Rs (Mueller et al., 2005; Meyerhof et al., 2011; Barretto et al., 2015), which are expressed within a specific population of taste sensory cells. Activating these taste cells either by genuine ligands or through optogenetics, triggers aversive reactions (Chen et al., 2011). By extension, bitter sensation is inferred in other animals, even in insects, since the activation of specific taste cells triggers aversive reactions often associated with feeding and serves to protect individuals from accidental ingestion of noxious molecules.

Toxic molecules are used in numerous species of all taxon including plants, animals, insects and microorganisms as a defense against their predators (Berenbaum, 1995; Skelhorn and Rowe, 2009). Such molecules encompass a bewildering array of chemical structures (Lunceford and Kubanek, 2015). Many of them are toxic to the consumer, and a number of them are deterrent or repellent (Kool, 2005). For consumers, it makes sense to be able to detect protected preys and to avoid feeding from sources contaminated with toxic or noxious molecules. Animals which exploit resources with low quantities of toxic molecules tend to lose their bitter receptors (Li and Zhang, 2014) as in whales (Feng et al., 2014) or vampire bats (Hong and Zhao, 2014). Specialist animals tend to have low numbers of bitter receptors while generalist animals tend to have more of them (McBride, 2007; McBride and Arguello, 2007). There are exceptions to this general hypothesis: for example, the silkworm *Bombyx mori* is an absolute specialist as it feeds and develops exclusively on leaves of the mulberry tree but its repertoire of taste receptors shows an expansion of bitter receptors (Wanner and Robertson, 2008). Inversely, the honeybee *Apis mellifera* which is a generalist, has a low number of gustatory receptors (Robertson and Wanner, 2006). These contradictions may resolve if one wants to consider not the chemistry of the molecules, but their biological role. For *B. mori*, it is possible that the expansion of gustatory receptors allow them to recognize secondary compounds associated with their specific host plant. For *A. mellifera*, it is possible that their food resource has a composition that limits the risks of being exposed to noxious molecules.

These observations suggest nevertheless that all organisms have evolved a taste modality that allows them to detect and to avoid molecules which represents a potential danger. This taste modality is defined both by an ensemble of taste receptor genes that define a “bitter” space, and by populations of receptor cells expressing members of this family of receptors. In this paper, we want to review recent evidence drawn mostly from our own experience in *Drosophila* that cells sensitive to bitter compounds react to classes of molecules important in different behavioral contexts, and stress that bitter molecules also have an impact on the detection of other molecules detected through other taste modalities.

## Contact Chemoreception in *Drosophila* Adults

Taste detection in *Drosophila* adults involves external and internal contact chemoreceptive sensilla which are distributed all over the body, especially in the oral region (proboscis and hypo- and epipharyngeal organs of the anterior digestive tract), on the legs, and on the front margins of the wings (Stocker, 1994; Shanbhag et al., 2001; Isono and Morita, 2010). Contact chemoreceptive sensilla have a pore at their tip, while olfactory sensilla have tiny pores all over the shaft (Altner and Prillinger, 1980; Stocker, 1994). Most of these taste sensilla house four gustatory neurons and a mechanosensitive neuron (Shanbhag et al., 2001). Some proboscis taste sensilla house only two taste neurons (Hiroi et al., 2004), while taste pegs which are located in rows between and on the lateral sides of the six pseudotracheal rows of the proboscis, house only one (Shanbhag et al., 2001). The cellular organization of these sensory units with bipolar sensory cells and three types of accessory cells, is very similar to that of olfactory sensilla found on the antenna and the maxillary palps. However, while olfactory receptors neurons converge into glomeruli in the antennal lobe, taste receptor neurons project into neuropiles associated with each body segment and appendage (de Bruyne and Warr, 2006; Kwon et al., 2014), thus combining a chemotopic and a somatotopic map (Wang et al., 2004), whereas in other insects, either a clear somatotopic map exists as in *Schistocerca gregaria* (Newland et al., 2000) and *Periplaneta americana* (Nishino et al., 2005), or not as in *Phormia regina* (Edgecomb and Murdock, 1992).

Since the initial discovery of a family of putative gustatory receptor proteins (Clyne et al., 1999), continuous progresses have been made in elucidating molecular elements which enable gustatory receptor neurons (GRNs) to detect external chemicals. In *Drosophila melanogaster*, this family includes 60 genes which encode for 68 receptor proteins (Clyne et al., 2000; Dunipace et al., 2001; Scott et al., 2001; Robertson et al., 2003). These receptors are expressed in GRNs but also in other tissues such as the digestive tract, reproductive organs and epidermal cells on the abdomen (Park and Kwon, 2011a,b), into the brain (*Gr43a* and *Gr64a*; Miyamoto et al., 2012; Miyamoto and Amrein, 2014; Fujii et al., 2015), into the antenna either as receptors to CO_2_ into specific sensilla (*Gr21a* and *Gr63a*; Jones et al., 2007; Yao and Carlson, 2010) or into olfactory neurons (*Gr5a*, *Gr64b* and *Gr64f*; Fujii et al., 2015) or even into multidendritic epithelial cells on the abdomen (*Gr66a*; Dunipace et al., 2001; Shimono et al., 2009). While GRs are generally thought to be involved in the detection of chemicals, they have been also shown to be involved in the detection of temperature (Ni et al., 2013).

GRNs express also a number of other genes which directly affect their sensitivity and selectivity. First of all, membrane-bound ionotropic receptors have been shown to affect pheromone and salt detection (Benton et al., 2009; Zhang et al., 2013a; Koh et al., 2014; Stewart et al., 2015). Transient receptor channels like TRPA1 and *pain* are involved in the detection of aversive molecules (Al-Anzi et al., 2006; Kim et al., 2010; Kwon et al., 2010), and pickpocket channels modulate pheromone and salt detection (Liu et al., 2003, 2012; Lin et al., 2005; Cameron et al., 2010; Chen et al., 2010; Lu et al., 2012; Pikielny, 2012; Starostina et al., 2012; Thistle et al., 2012; Toda et al., 2012; Alves et al., 2014). Taste sensitivity and selectivity is also modulated by proteins found in the sensillum lymph around the neurons such as odorant binding proteins (Galindo and Smith, 2001; Shanbhag et al., 2001; Koganezawa and Shimada, 2002; Park et al., 2006; Jeong et al., 2013), chemosensory proteins like CheB (Xu et al., 2002; Park et al., 2006; Ben-Shahar et al., 2007, 2010; Starostina et al., 2009) and various enzymes such as sugar-hydrolyzing proteins (Bhavsar et al., 1983).

This impressive array of genes is by no means complete but the picture that emerges seems clearer when it comes to mapping their expression to specific populations of neurons. Earlier electrophysiological studies in *Drosophila* promoted the view that GRNs would fall in four functional categories, respectively sensitive to sugars, salt, bitter molecules and water (Fujishiro et al., 1984; Singh, 1997; Meunier et al., 2003). Many exceptions to this scheme were found in various insects, such as water-cells responding to sugars (Wieczorek and Köppl, 1978; Wieczorek, 1980), or salt cells responding to sugar or lactose (Schnuch and Hansen, 1990, 1992). The situation is even more confusing in phytophagous insects where establishing a terminology distinguishing prototypic cell types across species seems quite difficult (Chapman, 2003). This lead Bernays and Chapman (2001) to consider only two functional types of cells, called phago-stimulant and phago-deterrent.

**Figure 1:**
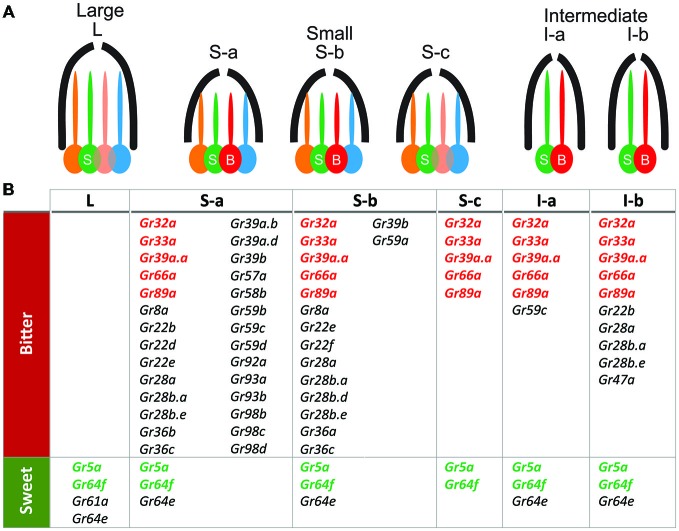
**Gr genes expressed in proboscis taste sensilla (after Weiss et al., 2011). (A)** Cellular composition of the different type of sensilla located on the external side of the proboscis. L-type sensilla house four neurons, one of which is sensitive to sugars (S). S-type sensilla house four neurons, including one sugar-sensitive neuron (S) and one sensitive to bitter (B); I-type sensilla house only two taste neurons (B and S). Each of these sensilla also include one mechanoreceptor neuron not represented here. **(B)** Table showing a map of the expression of the gustatory genes within the different types of sensilla and bitter-sensitive (bitter row) and sugar-sensitive (sweet row) neurons. This map was obtained by establishing GAL4 lines with the promoter of each of these gustatory genes to map the neurons which express these gustatory genes.

In flies at least two groups of sensory cells can be distinguished on the basis of the receptors they express (Figure 1): sugar-sensitive cells which co-express several gustatory genes such as *Gr5a, Gr64a-f* and *Gr61a* (Dahanukar et al., 2001, 2007; Scott et al., 2001; Thorne et al., 2004; Jiao et al., 2007; Slone et al., 2007; Weiss et al., 2011; Fujii et al., 2015), and bitter-sensitive cells which co-express several other gustatory genes such as *Gr66a*, *Gr33a* and *Gr93a* (Dunipace et al., 2001; Scott et al., 2001; Thorne et al., 2004; Moon et al., 2006; Weiss et al., 2011; Ling et al., 2014). Within these two categories, subtypes have been described both on the proboscis (Weiss et al., 2011) and on the legs (Ling et al., 2014), suggesting that flies may possess finer discrimination capabilities than currently thought (but see Masek and Scott, 2010).

It must be stressed that most of these observations rely upon the use of reporter genes using Gal4 or LexA enhancer trap systems (Brand and Perrimon, 1993; Lai and Lee, 2006; Miyazaki and Ito, 2010) as the level of expression of these genes is relatively low. This means that these data should be considered with caution. For example, the expression of *Gr64a* within sugar-sensitive GRNs has been recently challenged (Fujii et al., 2015) although previous studies had positively identified this gene as being expressed and involved in sugar perception in these GRNs (Dahanukar et al., 2007; Jiao et al., 2007, 2008). It is possible that these apparent discrepancies are not only due to limitations of the enhancer-trap approach, but also to differences of expression levels of these genes, depending on the genetic background or on the rearing conditions (Nishimura et al., 2012).

The current view is that several GR proteins are needed to make one functional receptor unit (Jiao et al., 2008; Lee et al., 2009, 2010). To be fully functional, a bitter receptor may need the co-expression of *Gr32a*, *Gr33a*, *Gr66a* (Moon et al., 2009; Lee et al., 2010) as well as of *Gr89a* and *Gr39a* which may represent “core-bitter *Grs*” (Weiss et al., 2011). Besides these core receptors, additional receptors may have a more specific role in the detection of particular chemicals such as GR59c for berberine, lobeline and denatonium (Weiss et al., 2011) and GR47a for strychnine (Lee et al., 2015). Sugar receptors may have a different set of core receptors (Dahanukar et al., 2001, 2007; Chyb et al., 2003; Jiao et al., 2007; Slone et al., 2007; Wisotsky et al., 2011; Ling et al., 2014; Yavuz et al., 2014; Fujii et al., 2015). This might explain why expressing individual bitter GRs into sugar-sensitive GRNs (and reversely) has failed so far (Lee et al., 2009; Montell, 2009; Isono and Morita, 2010).

The distinction between sugar- and bitter-sensitive taste cells is maintained in the way these cells project into the brain, in two non-overlapping areas at least in the suboesophageal ganglion (Wang et al., 2004; Marella et al., 2006; Miyazaki and Ito, 2010; Kwon et al., 2014; Harris et al., 2015). Activating one class of these receptors using ectopically expressed reporters triggers either appetitive or aversive behaviors (Wang et al., 2004; Marella et al., 2006; Hiroi et al., 2008; Harris et al., 2015).

The picture that emerges from these observations, however incomplete it might be, is that taste encoding in flies rests upon global categories or modalities such as appetitive or aversive (Thorne et al., 2004; Amrein and Thorne, 2005; Harris et al., 2015), in a way strikingly similar to what molecular studies have shown in vertebrates (Scott, 2005; Chandrashekar et al., 2006; Yarmolinsky et al., 2009; Chen et al., 2011; Liman et al., 2014; Barretto et al., 2015). The hypothesis that categories of receptors deal with different types of molecules inducing appetitive or aversive behaviors, does not match the view that emerged when recording from taste nerves in vertebrates, where no corresponding functional segregation could be made between fibers (Contreras and Lundy, 2000; Chen and Di Lorenzo, 2008; Frank et al., 2008). This latter encoding was called across fiber coding (Erickson, 2000, 2008a,b) as opposed to labeled lines coding. Actually, a similar inconsistency between peripheral recordings and the labeled line theory has been recently demonstrated in an insect, using multicellular recordings to monitor nerve activity and central responses in the suboesophageal ganglion of taste sensilla from the proboscis of *Manduca sexta* adults (Reiter et al., 2015). These opposed views (labeled lines *vs* across-fiber encoding) are difficult to reconcile (Scott and Giza, 2000; Smith et al., 2000; de Brito Sanchez and Giurfa, 2011) as each theory is missing elements for a complete proof (Fox, 2008).

## Direct Detection of Aversive Molecules

### Specific Taste Cells are Activated by Bitter Molecules

Adult flies respond to a number of alkaloids and aversive molecules by reducing their feeding intake. This can be observed using a number of different behavioral tests: by monitoring the proportion of flies that have fed upon diets containing colored dyes (Tanimura et al., 1982; Meunier et al., 2003), by measuring the quantity of liquid ingested by flies (Ja et al., 2007; Sellier et al., 2011) or by monitoring the proboscis extension upon stimulation of the legs or proboscis (Meunier et al., 2003; Masek and Scott, 2010). For example, quinine which is bitter to humans and to many animals including insects, inhibits feeding in a dose-dependent way starting at 10^−4^ M when mixed with 35 mM fructose in agar (Meunier et al., 2003). Behavioral inhibition of the proboscis extension reflex occurs even when berberine (another alkaloid) is presented on one leg while the other leg is stimulated with sugar (Meunier et al., 2003).

**Figure 2:**
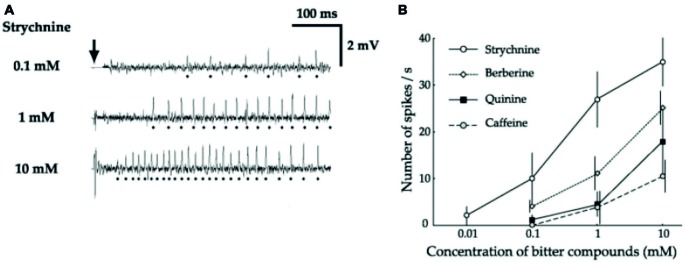
**Bitter-sensitive neurons are activated by bitter substances (from Hiroi et al., 2004). (A)** Sample recordings from I-type sensilla stimulated with strychnine at increasing concentrations (0.1 mM, 1 mM, 10 mM), showing that one cell is activated by strychnine. **(B)** Dose response curves showing the response of this cell to increasing concentrations of strychnine (empty circle), berberine (empty diamond), quinine (black square) and caffeine (empty circle and dotted line).

Electrophysiological recordings indicated that this behavioral inhibition is correlated with the activation of specific cells, present in some sensilla of the legs (Meunier et al., 2003) and on the proboscis (Figure 2; Hiroi et al., 2004; Sellier, 2010; Sellier et al., 2011). Further observations coupled with selective expression of various reporter genes demonstrate that flies indeed have one class of cells responding to bitter compounds in a dose-dependent way. These cells co-express several gustatory receptors (up to 28; Weiss et al., 2011; Figure 1). These cells may also co-express receptors belonging to other classes, such as TRPA1 (Kim et al., 2010) or *painless* which confers them the capability to respond to aversive compounds such as wasabi (Al-Anzi et al., 2006), or even to respond to noxious temperature (Ni et al., 2013).

This population of cells which all express *Gr66a* on the proboscis, can be activated artificially, by expressing receptors responding to new stimuli such as capsaicin using the human vanilloid receptor VR1 (Marella et al., 2006), to light using the channel rhodopsin CHR2 (Zhang et al., 2007; Honda et al., 2014; French et al., 2015), or even to an odor, butyl acetate, using an olfactory receptor *Or22a* and *Orco* (Hiroi et al., 2008). These observations support the view that taste cells expressing gustatory receptors such as *Gr66a*, *Gr32a* and *Gr33a* detect a variety of bitter stimuli (Marella et al., 2006; Harris et al., 2015) and induce aversive behavioral responses such as feeding inhibition.

### Bitter-Sensitive Taste Cells are Activated by Sex-Aversive Molecules

While contact chemoreceptors located all over the body are generally considered to function as detectors of sugars, bitter compounds, water and even salt, the detection of sexual pheromones is thought to be orchestrated by a group of specialized contact chemoreceptive sensilla. The distribution of these specialized sensilla is sexually dimorphic, whereby males have more taste sensilla on their legs (Nayak and Singh, 1983). During courtship, males go into several consecutive phases, one of which involves tapping on the abdomen of the females with their front legs (Spieth, 1974; Greenspan and Ferveur, 2000; Yamamoto and Koganezawa, 2013). Cobalt stainings showed that neurons from leg taste sensilla project differently in males than in females (Possidente and Murphey, 1989). This situation is confirmed by the fact that pheromone detection by contact involves numerous molecular elements apparently not related to bitter-tasting such as CheB proteins (Xu et al., 2002; Park et al., 2006), *ppk23, ppk25 and ppk29* DEG/Na channels (Lu et al., 2012; Pikielny, 2012; Thistle et al., 2012; Toda et al., 2012; Vijayan et al., 2014), gustatory receptors like *Gr39a*, *Gr32a* and *Gr68a* (Miyamoto and Amrein, 2008; Moon et al., 2009; Koganezawa et al., 2010; Wang et al., 2011; Watanabe et al., 2011), and ionotropic receptors (Koh et al., 2014).

**Figure 3:**
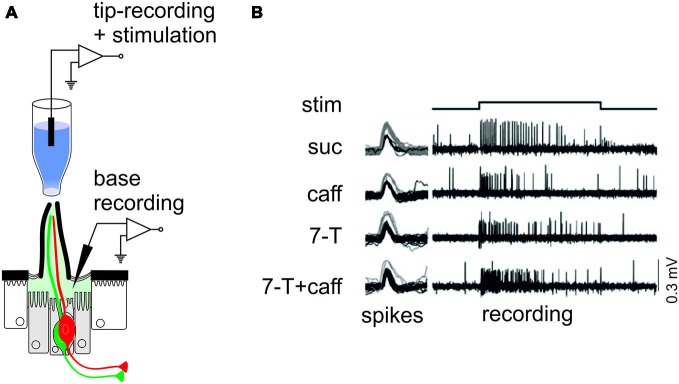
**Bitter-sensitive cells respond also to inhibitory sexual pheromones. (A)** Diagram showing the two electrodes configuration used to record extracellular activities from taste sensilla of *Drosophila*. In all cases, a glass capillary containing the stimulus is used to cap the tip of a gustatory sensillum. If the stimulus is water-soluble, the stimulus electrode can contain an electrolyte and can be used to record electrical signals from the neurons within the sensilla. If the stimulus is lipophilic, the stimulus electrode which contains paraffin oil with the ligand, is no longer conductive and we use another electrode, for example a fine tapered tungsten rod, inserted at the base of a sensillum. **(B)** Sample recordings obtained from an I-type sensillum on the proboscis of *Drosophila* using a tungsten recording electrode, and stimulating either with sucrose (suc), caffeine (caff), 7-tricosene (7-T) or a mixture of 7-tricosene and caffeine (reproduced from Lacaille et al., 2007).

However, very few studies have considered the wiring of these pheromone-sensitive cells, even though male-to-male detection is affected when “bitter” gustatory receptors such as *Gr32a* and *Gr38a* are inactivated (Miyamoto and Amrein, 2008; Moon et al., 2009). The involvement of *Gr32a* and *Gr38a* in pheromone detection is thought to be an indication that these *Grs* are obligatory co-receptors (Miyamoto and Amrein, 2008; Moon et al., 2009), in the same way as *Orco* (formerly known as *Or83b*) is an obligatory co-receptor in olfaction (Larsson et al., 2004). However, there is an even simpler explanation of the mixed roles of these *Grs* in the detection of pheromones and of bitter compounds, which is that aversive pheromones and bitter compounds may activate the same cells. We demonstrated on taste sensilla of the proboscis, that the same neuron responds both to caffeine and to 7-tricosene (7-T), which is a male inhibitory sexual pheromone (Figure 3). We further demonstrated that 7-T inhibits feeding while caffeine, berberine or quinine inhibit courtship (Lacaille et al., 2007). The simplest explanation of these observations is that the same neurons are used to detect different classes of signal, and that the central nervous system has limited capabilities to discriminate them. In other words, inhibitory pheromones taste “bitter” to flies.

Given the number of receptors expressed in this class of gustatory cells, *i.e.,* up to 28 *Grs*, TRP channels and IRs, it is likely that we have not yet found all the ligands to which bitter-sensitive cells respond. While most substances tested so far belong either to chemicals which are bitter to humans such as plant-derived compounds and artificial molecules like denatonium, or which play a role in intraspecific communication such as 7-T, it is tempting to speculate that bitter-sensitive taste neurons of flies also detect chemicals from their enemies, (predators, parasitoid insects or entomopathogens), or from their competitors such as bacteria or fungi. For example, grooming reactions can be induced in flies both by quinine and by extracts from the gram negative bacteria, *Escherichia coli* (Yanagawa et al., 2014), that belong to an entirely different category of chemicals than alkaloids and bitter molecules.

## Indirect Detection

While “bitter” molecules are detected by a specific class of gustatory cells, they might also interfere with the detection of molecules belonging to other modalities. Together with the activation of bitter-sensitive cells, sugar-sensing inhibition is considered as one of the major mechanisms by which plant secondary compounds exert antifeedant actions upon herbivores (Schoonhoven, 1982; Mitchell and Sutcliffe, 1984; Schoonhoven et al., 1992; Chapman, 2003). These inhibitions represent a “latent spectrum” as coined by Schoonhoven et al. (1992). Rather than being a curiosity or some kind of chemical artefact, we believe this mechanism represents an integral part of gustatory coding of bitter molecules in insects. Sugar-sensing inhibition by quinine for example has been observed very early in insects (Morita and Yamashita, 1959). In *Drosophila*, sugar-sensing inhibition (Siddiqi and Rodrigues, 1980), was described before bitter-sensitive cells were identified (Meunier et al., 2003).

Peripheral sugar-sensing inhibition seems a general phenomenon, as it occurs also in vertebrates (Akaike and Sato, 1976; Ogawa et al., 1997; Frank et al., 2005) and in other organisms such as leeches (Li et al., 2001). In vertebrates, sugar-sensing inhibition by quinine has been attributed to the direct inhibition of TRPM5 (Talavera et al., 2008), but also to interactions with G proteins (Naim et al., 1994), to K^+^ channels inhibition (Burgess et al., 1981) or even to the rapid entry into the cells inducing non-specific inhibition in taste cells (Peri et al., 2000). Thus far, no unitary mechanism explaining sugar-sensing inhibition by molecules such as quinine has been found. Bitter molecules may be detected either directly through a sensory receptor (not yet found), by interfering with the detection of sugar molecules via interaction with sugar receptors, or indirectly by interfering with or blocking various transduction elements.

**Figure 4:**
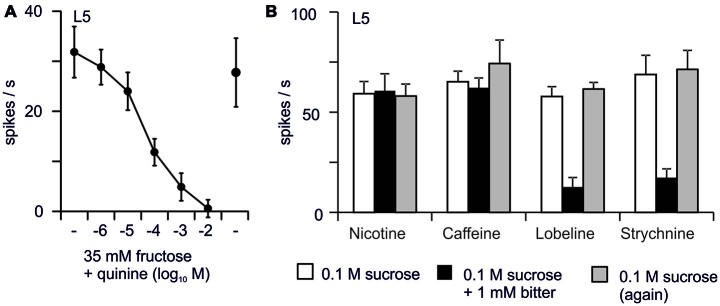
**Inhibition of the response to sugars by bitter chemicals (Sellier, 2010). (A)** Adding increasing concentrations of quinine to 35 mM fructose inhibits the firing activity recorded from L-type sensilla of the proboscis of *Drosophila*. **(B)** At the same molar concentration (1 mM), bitter chemicals differ in their power to inhibit the response to 0.1 M sucrose. Each point represents the average of 5–10 responses. Bars display SEM.

In *Drosophila*, sugar-sensing inhibition by bitter molecules can be demonstrated under at least two experimental situations. First, exposure to bitter chemicals may alter the detection of other tastants. For example, pre-exposing leg taste sensilla to 5 mM quinine during 10 s completely shuts down the response to sugar, and it takes 40 min to get a full recovery (Meunier et al., 2003). This inhibition might be due to a direct toxicity exerted upon nerve cells such as with vinblastine, colchicine (Matsumoto and Farley, 1978) or papain (Tanimura and Shimada, 1981), or it might be due to quinine molecules lingering in the sensillum lymph. Actually, as quinine is not prevalent in the environment of flies, they might miss proper degradation enzymes to clear the sensillum lymph. Secondly, bitter molecules may directly interfere with sugar detection (Sellier et al., 2011; French et al., 2015), either directly or indirectly, via an OBP (Jeong et al., 2013). Sugar-sensing inhibition differs between bitter chemicals (Figure 4; French et al., 2015), and between sugars (Schoonhoven, 1982; Schoonhoven and Liner, 1994; Martin and Shields, 2012). Given the enormous range in the chemical structures of “bitter” chemicals, it is likely that a variety of modes of action will be found.

In addition to peripheral sensory inhibition involving a direct interaction of bitter molecules with sugar sensitive cells, bitter chemicals may interfere with gustatory perception through other pathways. One mechanism could be through lateral interactions between sensory cells, for example through ephaptic inhibition as demonstrated for olfactory cells (Su et al., 2012). Such mechanism was not found in the taste sensilla tested so far (French et al., 2015), but non-synaptic interactions are definitely relevant for gustation. Another mechanism involves higher-order circuits, such as presynaptic inhibition of sugar sensing neurons by bitter-sensitive neurons through GABA receptors (Chu et al., 2014). Given the importance of the gustatory system in triggering or preventing feeding, we certainly expect modulations to occur at the level of the sensory neurons as well as in the central circuitry decoding this information. Recent observations made it clear that satiety has a strong effect on how odors are decoded (Ko et al., 2015), and how appetitive or bitter tastants trigger feeding reactions (Inagaki et al., 2014). Likewise, mating alters strongly female food preferences to proteins (Ribeiro and Dickson, 2010) and possibly to bitter chemicals as well.

## Future Prospects

All the data reported so far are compatible with the idea that bitter taste represents a well-defined taste modality which is different from sweet taste, at least when it comes to feeding. Bitter-sensitive cells are defined at a molecular level by the expression of a population of taste receptors, and activating these cells inhibits feeding. The behavioral inhibition is context-dependent, in that activating the same cells (on the proboscis) can either deter feeding or interfere with sex activities. This description is compatible with the view that insects may not be able to discriminate between different “bitter” molecules (Masek and Scott, 2010). Accordingly, the currently available data about how these neurons project in the central nervous system clearly indicate that bitter-sensitive neurons project to areas of the brain that are distinct from those where sugar-sensitive neurons project (Wang et al., 2004; Marella et al., 2006; Harris et al., 2015), maintaining the segregation observed at the periphery.

This might not be the last word of it, as sub-classes exist within the bitter modality (Weiss et al., 2011), and as taste neurons may encode bitter chemicals with different temporal codes (Glendinning et al., 2002, 2006) or even spatio-temporal codes (Reiter et al., 2015). However, even if one finds experimental evidence of rich encoding capabilities, so far, we are lacking clear behavioral evidences that flies can discriminate bitter molecules or bitter “categories”, independent of their concentration. Indications of such differences may come from looking more closely at different behaviors. For example, flies may prefer to lay eggs into food laced with bitter molecules (Yang et al., 2008; Schwartz et al., 2012; Dweck et al., 2013) instead of plain sugar (Yang et al., 2015), or into a medium rich in alcohol, especially if females were previously confronted with parasitoid wasps (Kacsoh et al., 2013, 2015). They might also change their natural preferences following larval exposure (Jaenike, 1982, 1983; Abed-Vieillard et al., 2014) or following the experience of others through social communication (Battesti et al., 2015). If not all “bitter” molecules are inducing aversive reactions in all behavioral contexts, this leaves open the possibility to test whether females can discriminate between different bitter molecules (but see Masek and Scott, 2010).

If the category “bitter” in flies regroup different shades or categories of bitterness, it seems to be pretty clear that the link between the noxiousness of molecules and their bitter taste is not a direct one. This lack of direct link has been clearly stated by Glendinning (1994, 2002, 2008), and has been experimentally tested in several phytophagous insects (Cottee et al., 1988; Usher et al., 1989; Bernays, 1990, 1991; Lee and Bernays, 1990; Bernays and Cornelius, 1992). This discrepancy between the intuitive role of bitterness to help avoiding intoxication and the lack of direct link between toxicity and bitterness should resolve if one considers aversive taste as a “correlation” established throughout evolution between a stimulus detected in the environment and a danger (or reduced fitness). One of the best examples for this comes from glucose-averse cockroaches (Silverman and Bieman, 1993) which avoid insecticide-treated diets, apparently through a mutation that allow resistant cockroaches to detect glucose (which is always associated with the insecticide) as a “bitter” molecule (Wada-Katsumata et al., 2013). Obviously, glucose is not toxic (Silverman, 1995; Silverman and Selbach, 1998), but it has become a signal for a toxic molecule in the environment.

**Figure 5:**
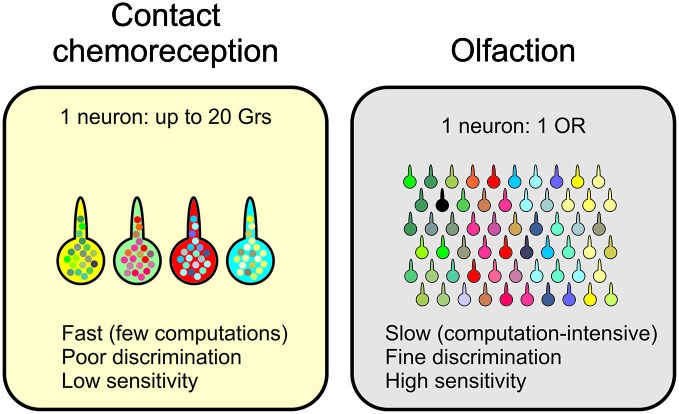
**Fundamental differences between olfaction and contact chemoreception in insects.** Although taste and olfactory sensilla have similar cellular compositions, the wiring of the neurons to the central nervous system and the number of different receptors expressed in each neuron is very different. These differences certainly impact the discriminative power and the speed at which information is processed.

Finally, it is striking to compare how information is analyzed in contact chemoreception and olfaction. Both systems are devoted to the detection of molecules in the external environment, using sensory receptors which are structured in a very similar way, with bipolar sensory cells enwrapped into accessory cells, sending dendrites into the sensillum lymph and their axon to the brain. However, the molecular logic and the wiring of the two systems are completely different. While the hedonic value of tastants seems to be determined already at the periphery with cells co-expressing a mosaic of receptors tuned to ligands pertaining to one or the other category, this distinction is less clear in olfaction (Knaden et al., 2012), as olfactory neurons express a very reduced set of receptors (Larsson et al., 2004; Goldman et al., 2005). This different structure probably imposes constraints on the functioning of the system, on its discriminative power, speed of decision and sensitivity threshold (Figure 5) as well as on its plasticity. Olfaction applies a relatively fixed array of filters on the external world, and decoding this grid of filters is done through a network of interconnected neurons at the level of the antennal lobes and then in the lateral horn and the mushroom bodies. This arrangement leaves room for plasticity in how information is decoded, taking into account experience and both internal and external environmental conditions. The gustatory system on the other hand appears more rigid with a bitter and a sweet modality defined by groups of gustatory receptors expressed in different categories of cells. Such a system does not seem to leave much space to plasticity as regards the hedonic value of molecules, except by modulating their impact by amplifying or decreasing their detection at the level of the central nervous system where a number of synaptic and neurohormonal regulations seem to occur, or directly at the level of the GRNs, which could modulate the level of expression of their different receptors (Zhang et al., 2013b).

## Conflict of Interest Statement

The authors declare that the research was conducted in the absence of any commercial or financial relationships that could be construed as a potential conflict of interest.
